# Efficacy and toxicity of aerosolised colistin in ventilator-associated pneumonia: a prospective, randomised trial

**DOI:** 10.1186/s13613-016-0127-7

**Published:** 2016-03-31

**Authors:** Sami Abdellatif, Ahlem Trifi, Foued Daly, Khaoula Mahjoub, Rochdi Nasri, Salah Ben Lakhal

**Affiliations:** Medical Intensive Care Unit, Tunis Faculty of Medicine, El Manar University, University Hospital Center La Rabta, Tunis, Tunisia

**Keywords:** Colistin, Aerosol, Ventilator-associated pneumonia, Nephrotoxicity, Intravenous

## Abstract

**Background:**

Cases of ventilator-associated pneumonia (VAP) due to multidrug-resistant (MDR) gram-negative bacilli (GNB) mainly *Acinetobacter baumannii*, *Pseudomonas aeruginosa* and enterobacteria are common in hospitalised patients of Tunisian intensive care units (ICUs). Parenteral colistin has been used for the therapy of VAP caused by MDR GNB at Tunisian hospitals over the past few years with a favourable clinical response. However, its use fell out of favour because of the reported drug-related nephrotoxicity and neurotoxicity.

**Objectives:**

To determine whether aerosolised (AS) colistin was beneficial and safe in therapy of gram-negative VAP.

**Methods:**

This was a randomised, single-blind study, in 149 critically ill adults who developed gram-negative VAP. Included patients were divided into two groups whether they received AS colistin (intervention group; *n* = 73) or intravenous (IV) colistin (control group; *n* = 76). AS colistin was given as 4 million units (MU) by nebulisation three times per 24 h. IV colistin was given as a loading dose of 9 MU followed by 4.5 MU two times per 24 h. Patients were followed during 28 days. Primary outcome was cure of VAP assessed at day 14 of therapy and defined as resolution of clinical signs of VAP and bacteriological eradication. Secondary outcomes were incidence of acute renal failure (ARF), mechanical ventilation length, ICU length of stay and 28-day mortality. Results were analysed based on intention-to-treat concept.

**Results:**

The patient’s baseline characteristics and distribution of pathogens VAP in both groups were similar. The clinical cure rate was 67.1 % in AS group and 72 % in IV group (*p* = 0.59). When administered in monotherapy or in combination, the AS regimen was as effective as IV regimen. Patients in AS group had significantly lower incidence of ARF (17.8 vs 39.4 %, *p* = 0.004), more favourable improvement of *P*/*F* ratio (349 vs 316 at day 14, *p* = 0.012), shortened time to bacterial eradication (TBE) (9.89 vs 11.26 days, *p* = 0.023) and earlier weaning from ventilator in ICU survivors with a mean gain in ventilator-free days of 5 days. No difference was shown in the length of stay and the 28-day mortality.

**Conclusion:**

Aerosolised colistin seems to be beneficial. It provided a therapeutic effectiveness non-inferior to parenteral colistin in therapy of MDR bacilli VAP with a lower nephrotoxicity, a better improvement of *P*/*F* ratio, a shortened bacterial eradication time and earlier weaning from ventilator in ICU survivors.

*Trial registration* ClinicalTrials.gov Identifier: NCT02683603

## Background

Ventilator-associated pneumonia (VAP) is the most common healthcare-associated infection causing morbidity and mortality [1]. Overall attributable mortality rates were between 5.8 and 27 % [1, 2]. In the past decade, multidrug-resistant (MDR) gram-negative bacteria (GNB) have become the focus of increased attention. Worldwide, as in Tunisia, there are growing threats to modern medicine from the emergence of MDR bacteria causing nosocomial infection. In Tunisian ICU, the most cases of VAP in patients are caused by GNB, predominantly *Acinetobacter baumannii*, *Pseudomonas aeruginosa* and *Klebsiella pneumonia* [3, 4]. Resistance of these MDR pathogens to β-lactams, including carbapenems, aminoglycosides and fluoroquinolones, was increasingly observed from patients with VAP [3, 4]. The virulence of such pathogens severely restricts viable therapeutic options. MDR *A. baumannii*, *P. aeruginosa* and *K. pneumonia* in Tunisian ICU are almost always susceptible to colistin, and parenteral colistin has been used in more than 90 % of nosocomial infections [5]. At our institution, over the past few years, the use of intravenous colistin was often associated with occurrence of side effects, mainly nephrotoxicity and neurotoxicity. Several studies on the therapy of MDR bacteria pneumonia with nebulised colistin revealed a trend of favourable use. Thus, the inhaled route may offer the benefit of an effective alveolar penetration with a low systemic diffusion [6]. Some reports with other antibiotics had approved the interest of such modality. Aerosolised aztreonam was recommended in cystic fibrosis since 2010 [7]. Tobramycin, aztreonam and colistin were assessed as adjunctive therapy with successful results [8–10].

The objective of the study was to determine whether aerosolised colistin was effective and safe in therapy of MDR bacilli VAP in comparison with intravenous colistin in a prospective randomised trial fashion.

## Methods

### Study design

This study was designed as a single-centre, prospective, randomised single-blind trial. It was conducted in a medical ICU of a tertiary care university teaching hospital during 25 months, from April 2013 to April 2015. The study protocol was approved by the ethics committee of the institutional review board of La Rabta Hospital.

### Patients and randomisation

All critically ill patients older than 18 years, with mechanical ventilation during more than 48 h, and who have presented a VAP, were eligible for study entry. Age <18 years, pregnancy and septic shock were considered as exclusion criteria. Patients who did not meet any exclusion criteria were randomly assigned into an intervention group and a control group (AS vs IV). Block randomisation was conducted by a random selection of computer-generated algorithm, the allocation sequence was followed by an independent statistician, and communicated to the investigator.

In the following cases: suspension of colistin (multisensitive strain imposing de-escalation, or a colistin-resistant strain), occurrence of a major side effect of inhaled route (severe bronchospasm or alveolar haemorrhage), decline in creatinine clearance below 10 ml/min in 48 h, occurrence of bacteraemia and/or septic shock, the patient should leave the trial protocol.

### Study intervention

Colistin used was colimycine^®^ powder (Sanofi Winthrop Industrie), i.e. colistimethate sodium—CMS. A flacon of 1 million units (MU) of colimycine^®^ = 80 mg of CMS = 33.3 mg of colistin base activity.

Included randomised patients were treated with an empirical anti-infective therapy combining imipenem and colistin, depending on our local bacterial ecology. According to the randomisation, patients were divided into two groups: intervention and control groups. The intervention group (AS group) received 4 million units (MU) of AS colistin by nebulisation for 30 min three times per day in addition to IV imipenem 1 g three times per day. Nebulisation was made via an ultrasonic vibrating plate nebuliser (Aeroneb Pro^®^ Aerogen Nektar Corporation, Galway, Ireland). This technique required specific settings in order to limit turbulence inspiratory flow. The specific settings were: a volume controlled mode with a tidal volume <8 ml/kg, respiratory rate at 12 cycles/min, I/E: 1/1 and an end inspiratory break >20 %.

The control group (IV group) received IV colistin as a loading dose of 9 MU during 60 min followed by 4.5 MU two times per day combined to IV impenem 1 g three times per day.

 According to anti-infective susceptibility results, a targeted therapy was started. If the strain was sensitive to other anti-infective drugs, colistin was combined with β-lactam or aminoglycoside or tygecyclin. If the isolated pathogen was a colistin-only susceptible, colistin was administered in monotherapy.

The treatment duration was maintained at least 14 days. After extubation, AS colistin dose was calculated according to a 40 % extra pulmonary deposition, as shown in experimental studies [11]. Thus, the prescribed dose was modified to 7 MU in the nebuliser chamber, equivalent to a delivered dose of 4.2 MU in the respiratory tract.

In case of renal insufficiency, the relay doses of IV colistin were modified according to the creatinine clearance as follows: 4.5 MU per day if 10 ml/min < clearance < 30 ml/min, and 4.5 MU per 48 h if clearance is less than 10 ml/min. The loading dose of 9 MU was maintained.

### Definitions and data collection

At study entry, demographic data, co-morbidities and the admission diagnosis were collected.

An episode of VAP was defined as a Clinical Pulmonary Infection Score (CPIS) of more than six [12, 13]. The investigators calculated the CPIS when the patient presented features suggesting a VAP such as fever, leukocytosis, purulent secretions, hypoxemia or radiological infiltrate. In case of the CPIS was higher than 6 points, the diagnosis of VAP was suspected and a tracheal aspirate was performed before colistin administration.

Bacteriological samples were performed on tracheal aspirate. A positive tracheal sample was defined as more than or equal to 10^6^ colony-forming units (CFU)/ml. Sensitivity to colistin was determined by the E test, and the isolated strain was considered sensitive when the minimum inhibitory concentration (MIC) was less than 2 mg/l. Microbiological eradication was defined as a negative culture, i.e. no pathogen isolated in tracheal aspiration. The oxygenation was assessed by the PaO2/FiO2 ratio, and its improvement was considered when the ratio exceeded 300.

The acute renal failure (ARF) was defined as an increase of plasma creatinine more than 1.5 times its base value.

### Outcome assessment

Patients were followed during 28 days. A physical examination (including temperature measurement and examination of tracheal secretions aspect) and a biological exploration including blood cells count, arterial blood gas and renal function were conducted every day. Chest X-ray and tracheal aspirate culture were performed every 3 days.

Therapeutic efficacy was assessed by the cure of VAP at the end of colistin therapy (day 14). The cure of VAP was defined as the resolution of clinical and biological signs of infection, i.e. a CPIS less than 6 and bacteriological eradication. Secondary outcomes were incidence of ARF, mechanical ventilation length, ICU length of stay and 28-day mortality.

### Post-therapy assessment

A clinical, biological and bacterial re-assessment was performed at day 7 of the anti-infective cure, by the CPIS calculation and renal function analysis. The tracheal aspirate was replaced by cyto-bacteriological sputum in case of ventilator weaning.

### Statistical analysis

We estimated that a sample size of 149 patients (AS, *n* = 73 and IV, *n* = 76) would provide a power of 80 % to demonstrate the non-inferiority of AS regimen compared with parenteral colistin with a lower toxicity in the intervention arm at a two-sided alpha error of 5 %. All statistical analyses were based on intention-to-treat principle.

Quantitative data were reported as mean ± SD and compared by the Student’s *t* test or the Mann–Whitney *U* test, as appropriate. Qualitative data were expressed as percentages and compared by the Chi-square or Fisher’s exact tests, as appropriate. The risk association measurement was expressed as odds ratio and performed by stratified analysis. Survival analysis was processed by the Kaplan–Meier survival curves and compared by the log-rank test. All tests were two-sided, and a *p* value <0.05 was considered to indicate statistical significance. The IBM SPSS Statistics 20 software was used for statistical analysis.

## Results

### Clinical and bacteriological characteristics

**Fig. 1:**
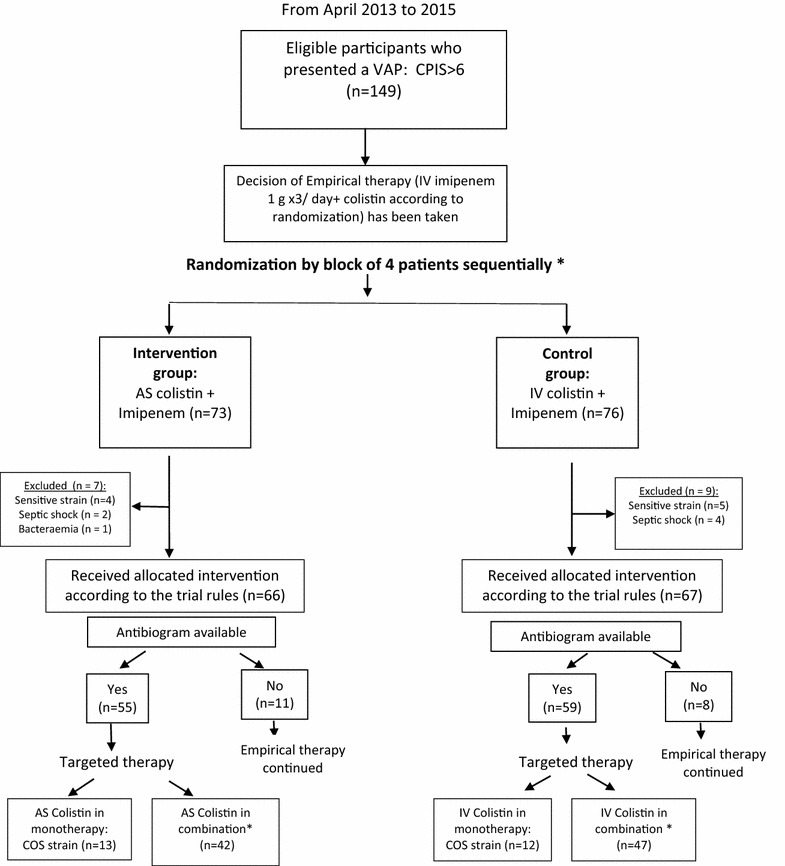
Patients’ flowchart. *VAP* ventilator-acquired pneumonia, *CPIS* Clinical Pulmonary Infection Score, *AS* aerosolised, *IV* intravenous, *COS* colistin-only susceptible. *The order of randomization was as follows: Block 1: AS, AS, IV, IV; Block 2: AS, IV, AS, IV; Block 3: IV, IV, AS, AS; Block 4: IV, AS, IV, AS. Details of combination are displayed in Table 1

One hundred and forty-nine patients have met our eligibility criteria. There were 73 evaluable patients in the AS group and 76 evaluable patients in the IV group. One hundred and thirty-three patients received allocated intervention and followed during the study period in accordance with the trial rules, while 16 patients did not receive allocated intervention. The two study groups were divided into two subgroups whether they received colistin alone or combined. The study flow diagram is shown in Fig. 1.

The demographic data, co-morbidities, admission diagnoses, the ICU stay before inclusion, pre-exposure to previous antibiotics and systemic antibiotics of the patients in both groups were not significantly different. When anti-biogram was available, in a median time of 3.6 days (the same period of empirical anti-infective therapy), colistin was continued either in combination (AS group; *n* = 60 and IV group; *n* = 64) or as monotherapy (AS group; *n* = 13 and IV group; *n* = 12). Carbapenems, β-lactam/β-lactamase inhibitors were the common antibiotics given in combination with colistin (Table 1).

**Table 1:** Baseline demographic and patients’ clinical characteristics

	AS colistin group (*n* = 73)	IV colistin group (*n* = 76)	*p* value
Age: age (mean + SD)	50 ± 16	53 ± 17	0.28
Sex ratio	2.31	1.81	0.49
SAPS II at inclusion	39 + 13	40 + 14	0.65
SOFA at inclusion	7.03 + 3.8	6.5 + 4.1	0.43
Reasons for admission [*n* (%)]			
Respiratory distress	32 (44 %)	29 (38 %)	
Neurological causes	23 (31.5 %)	26 (34 %)	
Metabolic disorders	11 (15 %)	15 (20 %)	
Intoxications	5 (7 %)	3 (4 %)	
Others	2 (2.5 %)	3 (4 %)	0.92
Length of stay before inclusion: days [mean + SD (median)]	11.28 ± 9 (9)	11.14 ± 8.8 (9)	0.49
Previous antibiotic use [*n* (%)]	49 (67 %)	46 (60 %)	1
Colistin in monotherapy [*n* (%)]	13 (17.8 %)	12 (15.7 %)	1
Co-administered antibiotics [*n* (%)]	60 (80.3 %)	64 (82 %)	0.62
β-lactams	32	37	1
Aminoglycosides	11	12	1
Quinolones or macrolides	5	6	0.79
Tygecyclin	8	8	0.58
Glycopeptides	8	6	
Iodinated contrast agent	14 (19 %)	17 (22.3 %)	0.68

*AS* aerosolised, *IV* intravenous, *SAPS II* Simplified Acute Physiology Score II, *SOFA* Sequential Organ Failure Assessment, *SD* standard deviation

**Fig. 2:**
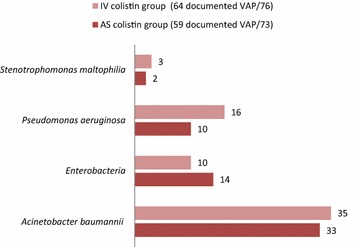
Microorganism’s distribution in study groups

In 123 (82.5 %) of the studied samples [AS group, *n* = 59 (81 %), and IV group, *n* = 64 (84.2 %)], a causing pathogen of VAP was isolated. The common causative bacteria isolates were *A. baumannii*, *P. aeruginosa* and *K. pneumonia*. There was no significant difference in microorganism’s distribution between the study groups: *p* = 0.65 (Fig. 2).

In case of VAP without isolated pathogen, the empirical therapy by imipenem/colistin according to randomisation was maintained.

### Therapeutic efficacy assessment

**Fig. 3:**
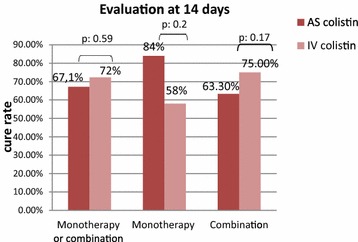
Cure rates between the study groups. The cure rates were shown when colistin was prescribed in monotherapy, in combination or both

Forty-nine patients of the AS group (67.1 %) had a favourable clinical outcome of VAP versus 72.3 % in IV group (*p* value = 0.59). Thus, efficacy of AS colistin in treatment of VAP was not inferior to IV colistin. Likewise, AS colistin was as effective as IV colistin; regardless it was administered as monotherapy or in combination. Rather, the observed cure rate with a monotherapy administration was greater than AS route but without statistical significance (Fig. 3).

### Assessment of efficacy items

All patients at inclusion have a radiological infiltrate among which the localised topography was more common than diffuse infiltration (64 % in AS arm and 68 % IV arm). Radiographic progression was observed 3 days later in 18 patients among AS group and 13 patients among IV group without significant difference (*p* = 0.41). The radiological clean-up was obtained in all patients with a favourable outcome.

**Fig. 4:**
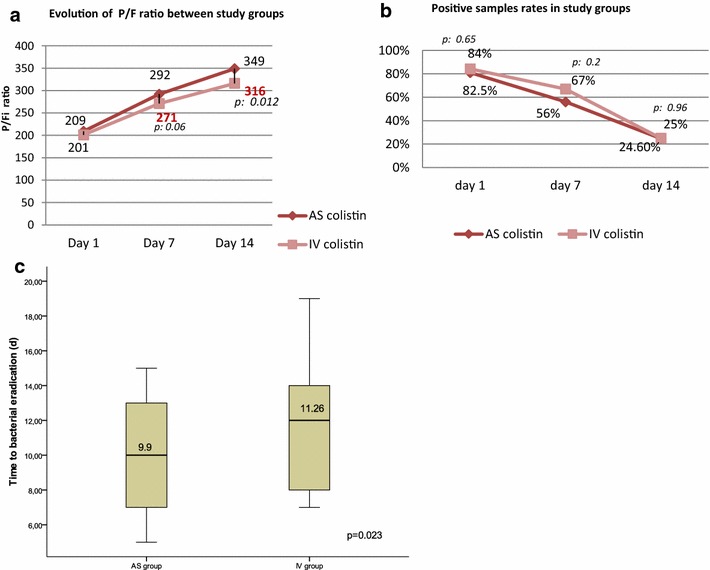
**a** Evolution of *P*/*F* ratio in both groups. **b** Evolution of positivity rate of bacteriological samples in both groups. **c** Time to bacterial eradication (TBE) between groups

In cured patients, the mean *P*/*F* ratio was significantly improved with AS colistin group at day 14 of colistin therapy. Microbiological outcomes did not differ in the negativity of samples, while, regarding the time to bacterial eradication (TBE), it was shortened by an average of 2 days in AS group (9.89 ± 2.7 vs 11.26 ± 3 days, *p* = 0.023) (Fig. 4).

### Systemic toxicity assessment

**Fig. 5:**
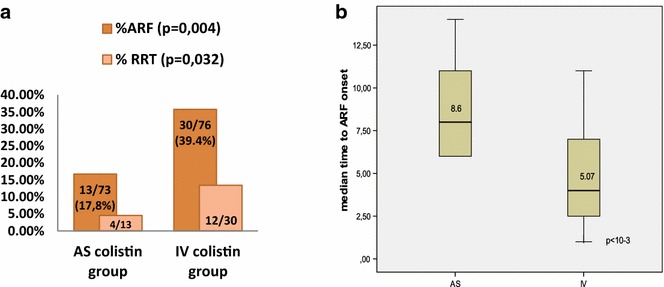
**a** Incidence of acute renal failure (ARF) and necessity of replacement renal therapy (RRT) in both groups. **b** Mean time to ARF onset in both groups

The incidence of acute renal failure (ARF) was significantly lower with AS administration. It occurred in 17.8 % of patients in the AS group compared with 39.4 % of patients in the IV group. Also, the replacement renal therapy (RRT) was less required with AS group (30.7 vs 40 %, *p* = 0.032). In the same way, the ARF onset was later in AS arm (8.69 v 5.07 days, *p* < 10^−3^) (Fig. 5). A stratified analysis on nephrotoxic agents (aminoglycosides, glycopeptides and iodinated contrast agents) showed a significant association between renal impairment and IV colistin (OR 2.79, 95 % CI [1.23; 6.32], *p* = 0.01).

A probable colistin-induced neurotoxicity occurred in 9 (12 %) and 7 (9.2 %) among AS and IV groups, respectively, without difference (*p* = 0.66). But, the causal relationship to colistin was difficult to establish because of other medications that could interfere (corticosteroids and neuromuscular blocking agents).

### Local toxicity assessment

Most of the patients tolerated nebulised colistin therapy well. A moderate bronchospasm was observed in 2.7 % of patients in the AS group. It was managed by bronchodilators without recurrence.

**Fig. 6:**
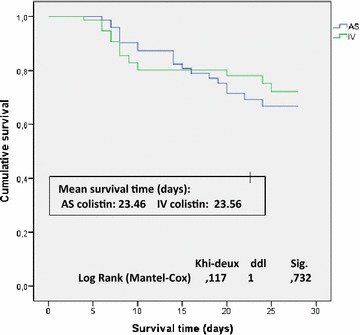
Survival analysis in study groups

No difference was observed in other secondary outcomes: mechanical ventilation length (13.8 ± 7 vs 16.5 ± 10, *p* = 0.083), ICU length of stay (25.9 ± 17 vs 26.07 ± 17, *p* = 0.9) and the all-cause mortality at 28 days (27.4 vs 23.7 %, *p* = 0.7) between AS and IV arms, respectively. Similarly, survival analysis did not differ (Fig. 6).

**Fig. 7:**
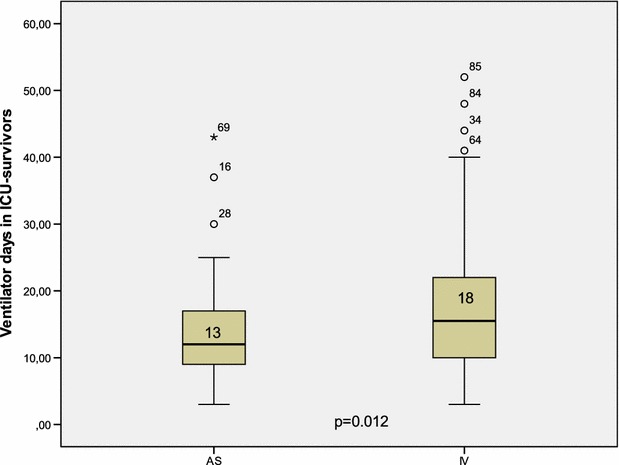
Duration of mechanical ventilation in study groups

Nevertheless, the results change with the ventilator days when only ICU survivors were included in the analysis (Fig. 7). Indeed, the weaning from ventilator was significantly earlier with AS group (13 vs 18 days; *p* = 0.012) with a mean gain in ventilator-free days of 5 days.

### Post-therapy assessment

Ninety patients were re-assessed at 7 days after colistin therapy (AS group; *n* = 46 and IV group; *n* = 44). Thirty-one patients died before this time, and 12 patients lost to follow-up.

**Fig. 8:**
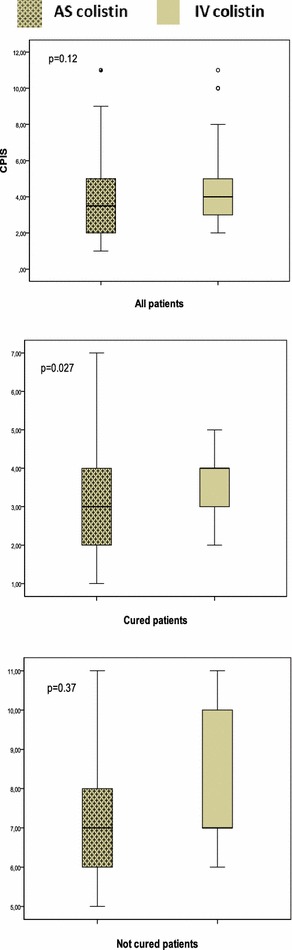
Post-therapy assessment of Clinical Pulmonary Infection Score (CPIS) in study groups

No difference in CPIS was shown in both groups (3.8 vs 4.5, *p* = 0.12). In cured patients, the CPIS was more enhanced with AS group (3.02 vs 3.68, *p* = 0.027) (Fig. 8). In case of clinical failure, the antibiotic therapy was prolonged or modified.

Respiratory samples were positive in 13 cases. We diagnosed a persistent VAP in four cases and colonisation in nine cases. Identified pathogens were: *Pseudomonas* spp. and *Acinetobacter baumannii* that maintained susceptibility to colistin. Others were *Serratia*, *Providencia* and *Proteus*.

The renal impairment was reversible in 78 % of AS group and 54 % of IV group (*p* = 0.38).

## Discussion

To the best of our knowledge, the present study is among the few randomised trials if not the only to date that evaluates the efficacy and safety of aerosolised colistin compared with parenteral colistin and not as adjunctive therapy to intravenous colistin in patients with gram-negative VAP. We demonstrate that nebulised colistin was as effective as IV colistin in therapy of VAP caused by MDR bacteria regardless of its prescription in combination or monotherapy (67.1 vs 72 %, *p* = 0.59). The benefit of such modality was shown at several points: a significant lower incidence of nephrotoxicity (17.8 vs 39.4 %, *p* = 0.004), a greater improvement of *P*/*F* ratio, a faster time to pathogen eradication and an earlier weaning from ventilator in ICU survivors.

### Rationale for using aerosolised antibiotics

The rationale for inhaling antibiotics is to maximise drug delivery to the target site of infection (i.e. the airways) and limit the potential for systemic side effects [14]. A major therapeutic advance of such modality took place in patients with cystic fibrosis chronically colonised with *P. aeruginosa* [7, 8, 15, 16]. Inhaled antibiotics were evaluated also in non-cystic fibrosis bronchiectasis with clinical benefits [17, 18]. In the experimental study of Lu et al. [11], colistin was found undetected in the lung tissue after intravenous infusion, while after nebulisation, peak lung tissue concentrations were significantly higher in the lung segments. The enhancement of the local bactericidal activity and the low systemic toxicity were also reported in a several experimental studies [6, 11, 19].

### Clinical benefits of aerosolised colistin in MDR bacteria VAP

 The majority of studies focused on inhaled colistin evaluated the efficacy and safety of nebulised colistin as adjunctive therapy to intravenous colistin compared with IV colistin [20–24] or compared with other antibiotics such as β-lactams [9], doxycycline [25] or tygecyclin [26]. The key results of these studies, separately or included in meta-analysis, were concordant in the beneficial effect of inhaled colistin without increasing the nephrotoxicity risk [20–24]. In our study, aerosolised colistin was as effective as intravenous colistin in therapy of MDR bacilli VAP. Moreover, when analysing singly the items of the primary outcome, we find an improvement of oxygenation (i.e. *P*/*F* ratio) and a faster bacterial eradication time. A similar result was reported by Polat et al. [27] in a paediatric intensive study that found a shorter median bacteriological eradication within 3 days when inhaled colistin was combined with IV colistin. The meta-analysis of Valachis et al. [28] reported a statistically significant improvement in clinical response and microbiological eradication (OR 1.57; 95 % CI [1.14–2.15]; p = 0.006 and OR 1.61; 95 % CI [1.11–2.35]; *p* = 0.01, respectively). These findings were not coherent with those of the meta-analysis of Gua et al. [29] that did not show a difference in the microbiological response (OR 1.29, CI [0.63–2.63], *p* = 0.48).

The administration of colistin aerosol via a pneumatic Aeroneb^®^ nebuliser requires some ventilator settings in order to maximise the intra-alveolar deposition. These conditions might participate in a better alveolar recruitment and therefore may interfere with the improvement of *P*/*F* ratio in the intervention arm.

### Systemic toxicity of inhaled colistin

Two types of toxicity, namely nephrotoxicity and neurotoxicity, have been reported with the uses of colistin. A systematic review of the toxicity of polymyxin revealed that in the old literature, incidences of both toxicities were reported to be considerably high, while new evidence shows less toxicity than previously reported although the definition of nephrotoxicity was not standardised between the studies [30]. Renal toxicity of colistin has been described mainly with the intravenous route at high doses [31–33]. No increase of renal toxicity risk was reported in the studies that tested inhaled colistin as adjunctive therapy [20–24]. Likewise, in the study of Lu et al. [9] that compared AS colistin versus β-lactams, no difference was reported between renal toxicity’s incidences (12 vs 8 %, *p* = 0.48). Else, it was shown that colistin trough plasma concentration significantly increased between days 2 and 3, suggesting colistin accumulation with time as a result of slow systemic passage through the alveolar–capillary membrane [9].

Our findings asserted the hypothesis of low systemic diffusion of inhaled colistin. Indeed, we observed a significant higher incidence of ARF, a more frequent requirement of RRT and an earlier time to onset of ARF with parenteral administration.

The concurrent administration of nephrotoxic drugs, hypovolemia or shock, and severity of illness may increase the likelihood of the development of ARF. In the current study, the stratified analysis on exposure to nephrotoxic drugs showed that the association of IV colistin/IRA was independent of these factors (OR 2.81, CI 95 % [1.24–6.38], *p* = 0.012).

Overall incidence of neurotoxicity related to colistin use is less than the nephrotoxicity. Earlier studies reported paresthesias in about one-fourth of patients receiving colistin, with few case reports of neuromuscular blockade or apnea while recent studies did not report any significant neurotoxicity [10, 34–36]. Neurotoxicity is also dose-dependent and may be triggered by the presence of risk factors like the presence of hypoxia, co-administration of muscle relaxant, narcotics, sedatives or steroids. In our study, a neurotoxicity induced by colistin was probably observed in 9 (12 %) and 7 (9.2 %) among AS and IV groups, respectively. The only causality of colistin was uncertain given the co-administration of other neurotoxic drugs (narcotics, sedatives, steroids).

### Local toxicity of inhaled colistin

Colistin aerosol inhalation therapy is generally well tolerated with few reported side effects like throat irritation, cough and bronchospasm, due to osmolality and preservatives within some of the solutions [37]. Among our AS group, 2.7 % of patients presented a moderate bronchospasm with a favourable evolution.

### Impact on morbidity and mortality

Another benefit of inhaled colistin in addition to the renal safety was revealed in our series: the gain of 5 days on ventilator-free days in ICU survivors. That result was the main consequence of the rapidest bacterial eradication time and the better improvement of the *P*/*F* ratio in the AS group.

No differences were revealed in the other secondary outcomes: the length of stay and 28-day mortality. These results were coherent with those of several meta-analyses [28, 29, 38].

Lu et al. reported a prolonged ICU length of stay and a prolonged duration of ventilation in the aerosol group [9].

### Acquisition of colistin resistance

It has been reported that prolonged use of IV colistin predisposes to VAP caused by pandrug-resistant bacteria, probably related to colistin poor lung tissue penetration and low concentrations at the infection site [39, 40], whereas nebulised colistin with a higher tissue concentrations in infected lung regions prevent selection of resistant strains [11]. On the other hand, acquisition of colistin resistance or increase in MICs was also reported and likely due to the incomplete destruction of the bronchial epithelium by colistin nebulisations and to production of a biofilm that constitute a protective space for bacteria and facilitate selection of resistant mutants [41, 42]. Low rates of acquisition of resistance were reported in recent studies [9, 29, 43]. As well, the current study did not detect an emergence of strains resistant to colistin acquisition. Yet, this finding must be interpreted reservedly because of the short follow-up in our series.

### Methodological limitations

The main limitation was the non-double-blind design of the trial protocol. Another pharmacologic limit was the absence of plasmatic dosages of colistin. These dosages could preferentially assert our clinical and biological results of the low systemic diffusion of the inhaled route.

## Conclusion

We conclude that the use of inhaled colistin seems to be beneficial in therapy of MDR bacilli VAP. Therapeutic effectiveness of such regimen was as effective as parenteral colistin. Further, it provided several benefits: a renal safety, a better improvement of *P*/*F* ratio, a shortened bacterial eradication time and an earlier weaning from ventilator in ICU survivors.

We suggest the regimen of aerosolised colistin as the first-line therapy in VAP due to MDR bacilli outside a septic shock and/or bacteraemia.

## Abbreviations

VAP: ventilator-associated pneumonia
MDR: multidrug resistant
GNB: gram-negative bacilli
IV: intravenously
AS: aerosolised
CPIS: Clinical Pulmonary Infection Score
MU: million units
Ml/min: millilitre/minute
CFU: colony-forming units
MIC: minimum inhibitory concentration
PaO_2_: arterial oxygen pressure
FiO2: inhaled oxygen fraction
TBE: time to bacterial eradication
ICU: intensive care unit
SD: standard deviation
COS: colistin-only susceptible
SAPS II: Simplified Acute Physiology Score II
SOFA: Sequential Organ Failure Assessment
MV: mechanical ventilation
ARF: acute renal failure
RRT: replacement renal therapy
